# *N*-[(*R*)-(6-Bromo-2-meth­oxy­quinolin-3-yl)(phen­yl)meth­yl]-*N*-[(*S*)-1-(4-meth­oxy­phen­yl)eth­yl]-2-(piperazin-1-yl)acetamide

**DOI:** 10.1107/S1600536811040955

**Published:** 2011-10-12

**Authors:** Lei Yuan, Rui Wang, Chang-Yi Li, Zhi-Qiang Wang, Tie-Min Sun

**Affiliations:** a Key Laboratory of Original New Drug Design & Discovery, Ministry of Education, College of Pharmaceutical Engineering, Shenyang Pharmaceutical University, Shenyang 110016, People’s Republic of China; b School of Pharmacy, Shanghai University of Traditional Chinese Medicine, Shanghai 201203, People’s Republic of China; c Tai’an Hospital of Chinese Medicine, Pharmacy Department, Tai’an 271000, People’s Republic of China

## Abstract

In the title compound, C_32_H_35_BrN_4_O_3_, the piperazine ring exists in a chair conformation. The quinoline ring system is oriented at dihedral angles of 82.70 (17) and 19.54 (17)° to the phenyl and meth­oxy­phenyl rings, respectively. Weak inter­molecular C—H⋯π inter­actions are present in the crystal structure.

## Related literature

For the synthesis of other phamaceutically active derivatives through conventional and other synthetic routes, see: Andries *et al.* (2005 ▶); Gaurrand *et al.* (2006 ▶); Mao *et al.* (2007 ▶); Dalla Via *et al.* (2008 ▶). For related structures, see: Cai *et al.* (2009 ▶); Petit *et al.* (2007 ▶).
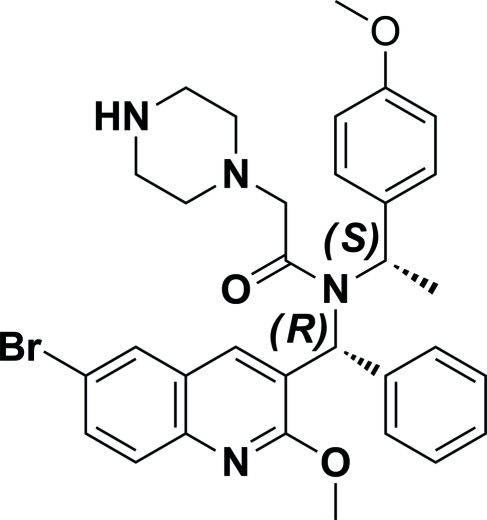


## Experimental

### 

#### Crystal data

C_32_H_35_BrN_4_O_3_
*M* *_r_* = 603.55
Orthorhombic, 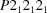

*a* = 9.9738 (9) Å
*b* = 10.9397 (10) Å
*c* = 27.910 (3) Å
*V* = 3045.3 (5) Å^3^
*Z* = 4
Mo *K*α radiation
μ = 1.39 mm^−1^
*T* = 293 K
0.26 × 0.21 × 0.13 mm

#### Data collection

Bruker APEXII diffractometer
Absorption correction: multi-scan (*SADABS*; Sheldrick, 2004 ▶) *T*_min_ = 0.712, *T*_max_ = 0.835
18937 measured reflections
5999 independent reflections
4518 reflections with *I* > 2σ(*I*)
*R*_int_ = 0.035

#### Refinement

*R*[*F* ^2^ > 2σ(*F* ^2^)] = 0.056
*wR*(*F* ^2^) = 0.170
*S* = 1.02
5999 reflections
361 parameters
H-atom parameters constrained
Δρ_max_ = 0.34 e Å^−3^
Δρ_min_ = −0.56 e Å^−3^
Absolute structure: Flack (1983 ▶), 2585 Friedel pairs
Flack parameter: 0.011 (12)

### 

Data collection: *APEX2* (Bruker, 2004 ▶); cell refinement: *SAINT* (Bruker, 2004 ▶); data reduction: *SAINT*; program(s) used to solve structure: *SHELXTL* (Sheldrick, 2008 ▶); program(s) used to refine structure: *SHELXTL*; molecular graphics: *SHELXTL*; software used to prepare material for publication: *SHELXTL*.

## Supplementary Material

Crystal structure: contains datablock(s) global, I. DOI: 10.1107/S1600536811040955/xu5339sup1.cif

Structure factors: contains datablock(s) I. DOI: 10.1107/S1600536811040955/xu5339Isup2.hkl

Supplementary material file. DOI: 10.1107/S1600536811040955/xu5339Isup3.cml

Additional supplementary materials:  crystallographic information; 3D view; checkCIF report

## Figures and Tables

**Table 1:** Hydrogen-bond geometry (Å, °) *Cg* is the centroid of the C12–C17 phenyl ring.

*D*—H⋯*A*	*D*—H	H⋯*A*	*D*⋯*A*	*D*—H⋯*A*
C10—H10*A*⋯*Cg*^i^	0.96	2.69	3.639 (6)	170

Symmetry code: (i) 
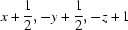
.

**Fig. 1.**
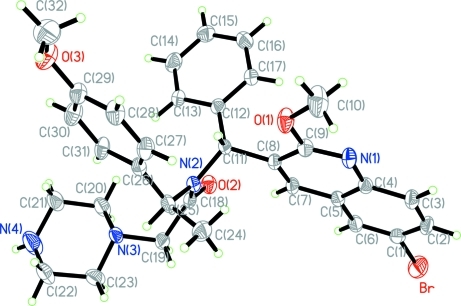
The molecular structure of the title compound, viewed along the a axis, showing 30%probability displacement ellipsoids and the atom- numbering scheme.

**Fig. 2.**
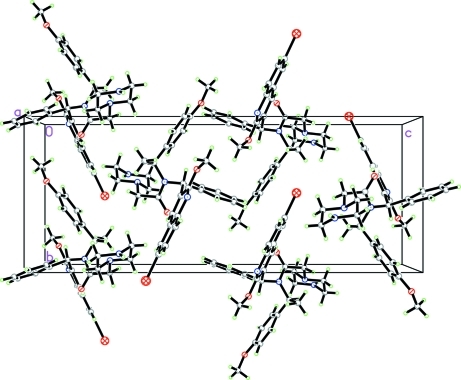
The crystal packing of the title compound.

## supplementary crystallographic information

### Comment

Most quinoline derivatives as a class of extremely important heterocyclic compounds are used in a wide array of synthetic and medical chemistry, such as antifungals, antituberculostatics, anticancer drugs and so on (Andries *et al.*, 2005). At the same time, the title compound is also a promising drug against tuberculosis. We synthesized this compound in order to get some more efficient antituberculosis drugs. To characterize our product, its single crystal structure was determined.

The structure of the title compound is shown in Fig. 1 and geometrical parameters are given in the archived CIF. In the title molecule, the bond lengths and angles are generally within normal ranges. The dihedral angles of aromatic rings are nearly in accordance with related structure TMC-207 (Petit *et al.*, 2007), which has been completed Phase II clinical, and will be marketed in 2012 as a kind of antituberculostatics drug.The dihedral angle between quinoline and phenyl ring [C12—C17] is 82.7°; The dihedral angle between quinoline and phenyl ring [C26—C31] is 19.5°; The dihedral angle between phenyl ring [C26—C31] and phenyl ring [C12—C17] is 78.6°; While the dihedral angle between phenyl and substituted quinolinyl group in TMC-207 is 97.4°, naphthalenyl and substituted quinolinyl group is nearly coplanar. On the other hand, the piperazidine ring exists in a chair conformationand. The bond angles also indicate sp^3^ hybridization nature of those atoms. No conventional hydrogen bonds were found at 293 (2)K for the title compound.

### Experimental

A solution of (1R,2S)-N-((6-bromo-2-methoxyquinolin-3-yl)(phenyl)methyl)-2- chloro-N-(1-(4-methoxyphenyl)ethyl)acetamide (1.0 mmol), piperazidine (1.1 mmol) and potassium carbonate (4.0 mmol) in acetonitrile was stirred for 4 h at 50°C.The reaction mixture was diluted with water (100 ml). The resulting precipitate was collected by filteration and purified on silica gel column (50% ethyl acetateu in petroleum ether) to give white powder (85.2% yield). A colorless crystalline solid was formed on slow evaporation of acetonitrile/methanol = 1:2 solution.

### Refinement

All H atoms were geometrically positioned (C–H 0.93—0.98 Å and N—H = 0.86 Å) and treated as riding, with U_iso_(H) = 1.5U_eq_(C) for methyl H atoms and 1.2U_eq_(C,N) for the others.

### Crystal data

**Table:**

C_32_H_35_BrN_4_O_3_	*F*(000) = 1256
*M**_r_* = 603.55	*D*_x_ = 1.316 Mg m^−^^3^
Orthorhombic, *P*2_1_2_1_2_1_	Mo *K*α radiation, λ = 0.71073 Å
Hall symbol: P 2ac 2ab	Cell parameters from 2354 reflections
*a* = 9.9738 (9) Å	θ = 2.0–25.0°
*b* = 10.9397 (10) Å	µ = 1.39 mm^−^^1^
*c* = 27.910 (3) Å	*T* = 293 K
*V* = 3045.3 (5) Å^3^	Block, colourless
*Z* = 4	0.26 × 0.21 × 0.13 mm

### Data collection

**Table:**

Bruker APEXII diffractometer	5999 independent reflections
Radiation source: fine-focus sealed tube	4518 reflections with *I* > 2σ(*I*)
graphite	*R*_int_ = 0.035
Detector resolution: 10.0 pixels mm^-1^	θ_max_ = 26.0°, θ_min_ = 1.5°
ω scans	*h* = −12→11
Absorption correction: multi-scan (*SADABS*; Sheldrick, 2004)	*k* = −13→13
*T*_min_ = 0.712, *T*_max_ = 0.835	*l* = −32→34
18937 measured reflections	

### Refinement

**Table:**

Refinement on *F*^2^	Secondary atom site location: difference Fourier map
Least-squares matrix: full	Hydrogen site location: inferred from neighbouring sites
*R*[*F*^2^ > 2σ(*F*^2^)] = 0.056	H-atom parameters constrained
*wR*(*F*^2^) = 0.170	*w* = 1/[σ^2^(*F*_o_^2^) + (0.109*P*)^2^] where *P* = (*F*_o_^2^ + 2*F*_c_^2^)/3
*S* = 1.02	(Δ/σ)_max_ = 0.001
5999 reflections	Δρ_max_ = 0.34 e Å^−^^3^
361 parameters	Δρ_min_ = −0.56 e Å^−^^3^
0 restraints	Absolute structure: Flack (1983), 2585 Friedel pairs
Primary atom site location: structure-invariant direct methods	Flack parameter: 0.011 (12)

### Special details

**Table:**

Geometry. All esds (except the esd in the dihedral angle between two l.s. planes) are estimated using the full covariance matrix. The cell esds are taken into account individually in the estimation of esds in distances, angles and torsion angles; correlations between esds in cell parameters are only used when they are defined by crystal symmetry. An approximate (isotropic) treatment of cell esds is used for estimating esds involving l.s. planes.
Refinement. Refinement of F^2^ against ALL reflections. The weighted R-factor wR and goodness of fit S are based on F^2^, conventional R-factors R are based on F, with F set to zero for negative F^2^. The threshold expression of F^2^ > 2sigma(F^2^) is used only for calculating R-factors(gt) etc. and is not relevant to the choice of reflections for refinement. R-factors based on F^2^ are statistically about twice as large as those based on F, and R- factors based on ALL data will be even larger.

### Fractional atomic coordinates and isotropic or equivalent isotropic displacement parameters (Å^2^)

**Table:**

	*x*	*y*	*z*	*U*_iso_*/*U*_eq_	
Br	1.16448 (6)	−0.50994 (6)	0.67941 (2)	0.0885 (3)	
O2	0.5985 (3)	−0.1119 (3)	0.63967 (10)	0.0483 (7)	
N2	0.6854 (3)	0.0788 (3)	0.64410 (10)	0.0371 (7)	
O3	0.5042 (4)	0.5855 (3)	0.55607 (14)	0.0717 (10)	
O1	1.0020 (3)	0.1519 (3)	0.56260 (13)	0.0601 (8)	
N3	0.4433 (3)	0.0798 (3)	0.71731 (11)	0.0407 (7)	
N1	1.1268 (3)	−0.0147 (3)	0.58380 (12)	0.0447 (8)	
C12	0.6723 (4)	0.0284 (3)	0.55538 (13)	0.0379 (8)	
C8	0.8885 (4)	−0.0074 (3)	0.60224 (12)	0.0366 (8)	
C4	1.1325 (4)	−0.1290 (3)	0.60612 (12)	0.0382 (8)	
C17	0.7336 (4)	−0.0184 (4)	0.51555 (13)	0.0463 (9)	
H17	0.8258	−0.0311	0.5157	0.056*	
C11	0.7584 (4)	0.0681 (3)	0.59812 (12)	0.0360 (8)	
H11	0.7878	0.1514	0.5905	0.043*	
C7	0.8947 (4)	−0.1195 (4)	0.62209 (13)	0.0419 (9)	
H7	0.8170	−0.1554	0.6340	0.050*	
C5	1.0190 (4)	−0.1841 (4)	0.62510 (13)	0.0408 (9)	
C25	0.7040 (4)	0.1975 (4)	0.67042 (14)	0.0438 (9)	
H25	0.6443	0.1949	0.6983	0.053*	
C6	1.0297 (4)	−0.3000 (4)	0.64702 (14)	0.0486 (10)	
H6	0.9539	−0.3391	0.6590	0.058*	
C26	0.6586 (4)	0.3032 (4)	0.64005 (13)	0.0414 (8)	
C2	1.2674 (4)	−0.2991 (4)	0.63264 (15)	0.0490 (10)	
H2	1.3500	−0.3375	0.6360	0.059*	
C19	0.5536 (4)	−0.0052 (5)	0.71269 (14)	0.0530 (11)	
H19A	0.5233	−0.0853	0.7229	0.064*	
H19B	0.6246	0.0200	0.7343	0.064*	
C3	1.2584 (4)	−0.1884 (4)	0.61003 (14)	0.0464 (10)	
H3	1.3348	−0.1522	0.5972	0.056*	
C16	0.6590 (4)	−0.0473 (4)	0.47472 (15)	0.0567 (11)	
H16	0.7017	−0.0794	0.4479	0.068*	
C9	1.0115 (4)	0.0395 (3)	0.58310 (14)	0.0414 (9)	
C13	0.5344 (4)	0.0471 (4)	0.55422 (15)	0.0482 (10)	
H13	0.4913	0.0802	0.5808	0.058*	
C27	0.7433 (4)	0.3932 (4)	0.62201 (16)	0.0522 (11)	
H27	0.8347	0.3882	0.6283	0.063*	
C29	0.5599 (4)	0.4968 (4)	0.58342 (16)	0.0514 (10)	
C31	0.5236 (4)	0.3155 (4)	0.62922 (17)	0.0496 (10)	
H31	0.4634	0.2582	0.6412	0.060*	
C28	0.6960 (4)	0.4898 (4)	0.59510 (17)	0.0554 (11)	
H28	0.7547	0.5504	0.5847	0.066*	
C18	0.6126 (4)	−0.0162 (4)	0.66139 (14)	0.0418 (9)	
C15	0.5229 (5)	−0.0284 (4)	0.47393 (16)	0.0564 (11)	
H15	0.4734	−0.0462	0.4466	0.068*	
C30	0.4758 (5)	0.4090 (4)	0.60162 (18)	0.0581 (12)	
H30	0.3845	0.4132	0.5950	0.070*	
C24	0.8467 (5)	0.2064 (5)	0.68981 (17)	0.0624 (12)	
H24A	0.8662	0.1354	0.7088	0.094*	
H24B	0.8550	0.2783	0.7093	0.094*	
H24C	0.9087	0.2110	0.6636	0.094*	
C1	1.1521 (5)	−0.3534 (4)	0.65043 (13)	0.0505 (10)	
C23	0.4107 (5)	0.0920 (6)	0.76822 (15)	0.0657 (14)	
H23A	0.4881	0.1228	0.7854	0.079*	
H23B	0.3881	0.0124	0.7813	0.079*	
C20	0.3260 (4)	0.0351 (5)	0.69106 (16)	0.0595 (12)	
H20A	0.3012	−0.0448	0.7032	0.071*	
H20B	0.3489	0.0261	0.6575	0.071*	
C14	0.4610 (5)	0.0170 (5)	0.51402 (16)	0.0605 (12)	
H14	0.3685	0.0276	0.5141	0.073*	
C22	0.2923 (5)	0.1794 (6)	0.77515 (19)	0.0723 (15)	
H22A	0.2710	0.1854	0.8090	0.087*	
H22B	0.3168	0.2602	0.7638	0.087*	
N4	0.1719 (5)	0.1351 (6)	0.74826 (18)	0.0963 (17)	
H4A	0.0942	0.1210	0.7604	0.116*	
C21	0.2101 (5)	0.1193 (7)	0.69568 (18)	0.0829 (19)	
H21A	0.1343	0.0867	0.6780	0.100*	
H21B	0.2330	0.1981	0.6820	0.100*	
C10	1.1213 (5)	0.1999 (5)	0.5407 (3)	0.0880 (19)	
H10A	1.1025	0.2788	0.5272	0.132*	
H10B	1.1504	0.1455	0.5158	0.132*	
H10C	1.1906	0.2073	0.5644	0.132*	
C32	0.5833 (7)	0.6817 (5)	0.5379 (2)	0.0825 (16)	
H32A	0.5281	0.7354	0.5191	0.124*	
H32B	0.6533	0.6488	0.5181	0.124*	
H32C	0.6222	0.7265	0.5640	0.124*	

### Atomic displacement parameters (Å^2^)

**Table:**

	*U*^11^	*U*^22^	*U*^33^	*U*^12^	*U*^13^	*U*^23^
Br	0.0962 (5)	0.0790 (4)	0.0903 (4)	0.0348 (3)	0.0057 (3)	0.0237 (3)
O2	0.0438 (15)	0.0432 (16)	0.0578 (16)	0.0021 (13)	0.0115 (13)	0.0067 (14)
N2	0.0342 (17)	0.0399 (16)	0.0371 (15)	0.0056 (14)	0.0051 (13)	−0.0013 (13)
O3	0.065 (2)	0.053 (2)	0.097 (2)	0.0068 (16)	−0.0065 (19)	0.0160 (18)
O1	0.0421 (16)	0.0471 (17)	0.091 (2)	0.0057 (14)	0.0201 (15)	0.0181 (16)
N3	0.0303 (16)	0.052 (2)	0.0395 (17)	0.0027 (14)	0.0058 (13)	−0.0062 (15)
N1	0.0298 (16)	0.0457 (18)	0.0586 (19)	0.0010 (14)	0.0049 (14)	−0.0059 (16)
C12	0.039 (2)	0.0334 (18)	0.0411 (18)	0.0038 (16)	0.0024 (16)	0.0054 (14)
C8	0.0315 (18)	0.0396 (19)	0.0386 (18)	0.0056 (16)	0.0041 (14)	0.0014 (16)
C4	0.036 (2)	0.040 (2)	0.0389 (19)	0.0040 (16)	−0.0022 (15)	−0.0102 (15)
C17	0.038 (2)	0.056 (2)	0.045 (2)	0.0102 (19)	0.0055 (16)	0.0024 (19)
C11	0.0310 (19)	0.041 (2)	0.0360 (19)	0.0076 (16)	0.0088 (15)	0.0030 (15)
C7	0.0311 (18)	0.050 (2)	0.045 (2)	0.0083 (17)	0.0079 (16)	0.0051 (17)
C5	0.042 (2)	0.044 (2)	0.0373 (19)	0.0103 (17)	0.0066 (16)	0.0013 (16)
C25	0.037 (2)	0.051 (2)	0.043 (2)	0.0068 (17)	0.0039 (16)	−0.0121 (17)
C6	0.050 (2)	0.049 (2)	0.046 (2)	0.014 (2)	0.0122 (19)	0.0105 (18)
C26	0.035 (2)	0.044 (2)	0.0448 (19)	0.0037 (18)	0.0023 (17)	−0.0102 (16)
C2	0.041 (2)	0.050 (2)	0.056 (2)	0.0195 (19)	−0.009 (2)	−0.015 (2)
C19	0.045 (2)	0.068 (3)	0.045 (2)	0.020 (2)	0.0138 (17)	0.019 (2)
C3	0.030 (2)	0.053 (2)	0.056 (2)	0.0023 (18)	−0.0011 (17)	−0.016 (2)
C16	0.056 (3)	0.066 (3)	0.048 (2)	0.006 (2)	0.003 (2)	−0.011 (2)
C9	0.038 (2)	0.039 (2)	0.047 (2)	0.0015 (17)	0.0038 (17)	0.0021 (16)
C13	0.037 (2)	0.061 (3)	0.046 (2)	0.0158 (19)	0.0006 (18)	0.0003 (18)
C27	0.031 (2)	0.055 (3)	0.070 (3)	−0.0005 (19)	0.002 (2)	−0.003 (2)
C29	0.049 (2)	0.043 (2)	0.062 (2)	0.007 (2)	0.0042 (19)	0.001 (2)
C31	0.028 (2)	0.043 (2)	0.078 (3)	0.0025 (17)	0.0074 (19)	0.007 (2)
C28	0.043 (2)	0.045 (2)	0.079 (3)	−0.006 (2)	0.014 (2)	−0.001 (2)
C18	0.0315 (18)	0.049 (2)	0.0449 (19)	0.0122 (17)	0.0053 (16)	0.0067 (18)
C15	0.056 (3)	0.064 (3)	0.049 (2)	0.004 (2)	−0.011 (2)	−0.004 (2)
C30	0.037 (2)	0.050 (3)	0.087 (3)	−0.0009 (19)	−0.005 (2)	0.003 (2)
C24	0.051 (3)	0.074 (3)	0.062 (3)	0.008 (2)	−0.014 (2)	−0.012 (2)
C1	0.062 (3)	0.053 (2)	0.037 (2)	0.021 (2)	−0.0003 (19)	−0.0004 (17)
C23	0.045 (3)	0.109 (4)	0.043 (2)	0.000 (3)	0.002 (2)	−0.011 (3)
C20	0.043 (2)	0.079 (3)	0.056 (2)	0.001 (2)	0.0051 (19)	−0.024 (2)
C14	0.037 (2)	0.073 (3)	0.071 (3)	0.005 (2)	−0.005 (2)	0.006 (3)
C22	0.047 (3)	0.102 (4)	0.068 (3)	−0.002 (3)	0.011 (2)	−0.041 (3)
N4	0.059 (3)	0.143 (5)	0.087 (3)	0.010 (3)	0.013 (2)	−0.045 (3)
C21	0.043 (3)	0.141 (6)	0.065 (3)	0.025 (3)	−0.004 (2)	−0.032 (3)
C10	0.058 (3)	0.066 (3)	0.140 (5)	0.000 (3)	0.041 (3)	0.032 (3)
C32	0.112 (5)	0.051 (3)	0.085 (3)	−0.003 (3)	0.009 (4)	0.013 (3)

### Geometric parameters (Å, °)

**Table:**

Br—C1	1.898 (4)	C19—H19B	0.9700
O2—C18	1.217 (5)	C3—H3	0.9300
N2—C18	1.357 (5)	C16—C15	1.373 (6)
N2—C11	1.480 (4)	C16—H16	0.9300
N2—C25	1.504 (5)	C13—C14	1.379 (6)
O3—C29	1.353 (5)	C13—H13	0.9300
O3—C32	1.410 (6)	C27—C28	1.379 (6)
O1—C9	1.360 (5)	C27—H27	0.9300
O1—C10	1.437 (6)	C29—C30	1.373 (6)
N3—C19	1.446 (5)	C29—C28	1.398 (6)
N3—C23	1.464 (5)	C31—C30	1.366 (6)
N3—C20	1.464 (5)	C31—H31	0.9300
N1—C9	1.294 (5)	C28—H28	0.9300
N1—C4	1.399 (5)	C15—C14	1.371 (7)
C12—C17	1.368 (5)	C15—H15	0.9300
C12—C13	1.391 (5)	C30—H30	0.9300
C12—C11	1.533 (5)	C24—H24A	0.9600
C8—C7	1.346 (5)	C24—H24B	0.9600
C8—C9	1.433 (5)	C24—H24C	0.9600
C8—C11	1.543 (5)	C23—C22	1.531 (7)
C4—C5	1.388 (5)	C23—H23A	0.9700
C4—C3	1.418 (5)	C23—H23B	0.9700
C17—C16	1.398 (6)	C20—C21	1.483 (7)
C17—H17	0.9300	C20—H20A	0.9700
C11—H11	0.9800	C20—H20B	0.9700
C7—C5	1.429 (5)	C14—H14	0.9300
C7—H7	0.9300	C22—N4	1.497 (7)
C5—C6	1.412 (6)	C22—H22A	0.9700
C25—C26	1.503 (6)	C22—H22B	0.9700
C25—C24	1.526 (6)	N4—C21	1.526 (7)
C25—H25	0.9800	N4—H4A	0.8600
C6—C1	1.357 (6)	C21—H21A	0.9700
C6—H6	0.9300	C21—H21B	0.9700
C26—C31	1.387 (6)	C10—H10A	0.9600
C26—C27	1.392 (6)	C10—H10B	0.9600
C2—C3	1.368 (6)	C10—H10C	0.9600
C2—C1	1.387 (6)	C32—H32A	0.9600
C2—H2	0.9300	C32—H32B	0.9600
C19—C18	1.553 (5)	C32—H32C	0.9600
C19—H19A	0.9700		
			
C18—N2—C11	120.8 (3)	O3—C29—C30	117.3 (4)
C18—N2—C25	123.6 (3)	O3—C29—C28	124.7 (4)
C11—N2—C25	115.5 (3)	C30—C29—C28	118.0 (4)
C29—O3—C32	120.6 (4)	C30—C31—C26	122.3 (4)
C9—O1—C10	116.8 (3)	C30—C31—H31	118.8
C19—N3—C23	108.3 (3)	C26—C31—H31	118.8
C19—N3—C20	110.4 (3)	C27—C28—C29	120.1 (4)
C23—N3—C20	109.8 (3)	C27—C28—H28	120.0
C9—N1—C4	116.9 (3)	C29—C28—H28	120.0
C17—C12—C13	118.6 (4)	O2—C18—N2	122.9 (3)
C17—C12—C11	119.2 (3)	O2—C18—C19	118.9 (4)
C13—C12—C11	122.0 (3)	N2—C18—C19	118.1 (4)
C7—C8—C9	116.1 (3)	C14—C15—C16	119.1 (4)
C7—C8—C11	123.8 (3)	C14—C15—H15	120.5
C9—C8—C11	120.0 (3)	C16—C15—H15	120.5
C5—C4—N1	121.7 (3)	C31—C30—C29	121.3 (4)
C5—C4—C3	119.7 (4)	C31—C30—H30	119.4
N1—C4—C3	118.7 (3)	C29—C30—H30	119.4
C12—C17—C16	120.6 (4)	C25—C24—H24A	109.5
C12—C17—H17	119.7	C25—C24—H24B	109.5
C16—C17—H17	119.7	H24A—C24—H24B	109.5
N2—C11—C12	114.9 (3)	C25—C24—H24C	109.5
N2—C11—C8	113.0 (3)	H24A—C24—H24C	109.5
C12—C11—C8	112.2 (3)	H24B—C24—H24C	109.5
N2—C11—H11	105.2	C6—C1—C2	122.4 (4)
C12—C11—H11	105.2	C6—C1—Br	118.5 (3)
C8—C11—H11	105.2	C2—C1—Br	119.0 (3)
C8—C7—C5	120.9 (4)	N3—C23—C22	110.5 (4)
C8—C7—H7	119.5	N3—C23—H23A	109.5
C5—C7—H7	119.5	C22—C23—H23A	109.5
C4—C5—C6	119.5 (4)	N3—C23—H23B	109.5
C4—C5—C7	118.1 (4)	C22—C23—H23B	109.5
C6—C5—C7	122.4 (4)	H23A—C23—H23B	108.1
C26—C25—N2	110.6 (3)	N3—C20—C21	111.8 (4)
C26—C25—C24	115.6 (4)	N3—C20—H20A	109.3
N2—C25—C24	110.1 (3)	C21—C20—H20A	109.3
C26—C25—H25	106.7	N3—C20—H20B	109.3
N2—C25—H25	106.7	C21—C20—H20B	109.3
C24—C25—H25	106.7	H20A—C20—H20B	107.9
C1—C6—C5	119.1 (4)	C15—C14—C13	120.8 (4)
C1—C6—H6	120.5	C15—C14—H14	119.6
C5—C6—H6	120.5	C13—C14—H14	119.6
C31—C26—C27	116.2 (4)	N4—C22—C23	110.7 (4)
C31—C26—C25	119.4 (4)	N4—C22—H22A	109.5
C27—C26—C25	124.5 (4)	C23—C22—H22A	109.5
C3—C2—C1	119.4 (4)	N4—C22—H22B	109.5
C3—C2—H2	120.3	C23—C22—H22B	109.5
C1—C2—H2	120.3	H22A—C22—H22B	108.1
N3—C19—C18	114.9 (3)	C22—N4—C21	108.6 (4)
N3—C19—H19A	108.5	C22—N4—H4A	125.7
C18—C19—H19A	108.5	C21—N4—H4A	125.7
N3—C19—H19B	108.5	C20—C21—N4	110.4 (5)
C18—C19—H19B	108.5	C20—C21—H21A	109.6
H19A—C19—H19B	107.5	N4—C21—H21A	109.6
C2—C3—C4	119.9 (4)	C20—C21—H21B	109.6
C2—C3—H3	120.0	N4—C21—H21B	109.6
C4—C3—H3	120.0	H21A—C21—H21B	108.1
C15—C16—C17	120.4 (4)	O1—C10—H10A	109.5
C15—C16—H16	119.8	O1—C10—H10B	109.5
C17—C16—H16	119.8	H10A—C10—H10B	109.5
N1—C9—O1	118.8 (3)	O1—C10—H10C	109.5
N1—C9—C8	126.3 (4)	H10A—C10—H10C	109.5
O1—C9—C8	114.9 (3)	H10B—C10—H10C	109.5
C14—C13—C12	120.5 (4)	O3—C32—H32A	109.5
C14—C13—H13	119.7	O3—C32—H32B	109.5
C12—C13—H13	119.7	H32A—C32—H32B	109.5
C28—C27—C26	122.1 (4)	O3—C32—H32C	109.5
C28—C27—H27	118.9	H32A—C32—H32C	109.5
C26—C27—H27	118.9	H32B—C32—H32C	109.5

### Hydrogen-bond geometry (Å, °)

**Table:**

Cg is the centroid of the C12–C17 phenyl ring.

**Table:**

*D*—H···*A*	*D*—H	H···*A*	*D*···*A*	*D*—H···*A*
C10—H10A···Cg^i^	0.96	2.69	3.639 (6)	170.

Symmetry codes: (i) *x*+1/2, −*y*+1/2, −*z*+1.
